# Trends in Computer-Aided Manufacturing in Prosthodontics: A Review of the Available Streams

**DOI:** 10.1155/2014/783948

**Published:** 2014-04-08

**Authors:** Jaafar Abduo, Karl Lyons, Mohammed Bennamoun

**Affiliations:** ^1^School of Dentistry, Melbourne University, 720 Swanston Street, Carlton, Melbourne, VIC, 3010, Australia; ^2^School of Computer Science and Software Engineering, University of Western Australia, 35 Stirling Highway, Crawley, WA 6009, Australia; ^3^Department of Oral Rehabilitation, Faculty of Dentistry, University of Otago, 310 Great King Street, Dunedin 9054, New Zealand

## Abstract

In prosthodontics, conventional methods of fabrication of oral and facial prostheses have been considered the gold standard for many years. The development of computer-aided manufacturing and the medical application of this industrial technology have provided an alternative way of fabricating oral and facial prostheses. This narrative review aims to evaluate the different streams of computer-aided manufacturing in prosthodontics. To date, there are two streams: the subtractive and the additive approaches. The differences reside in the processing protocols, materials used, and their respective accuracy. In general, there is a tendency for the subtractive method to provide more homogeneous objects with acceptable accuracy that may be more suitable for the production of intraoral prostheses where high occlusal forces are anticipated. Additive manufacturing methods have the ability to produce large workpieces with significant surface variation and competitive accuracy. Such advantages make them ideal for the fabrication of facial prostheses.

## 1. Introduction

Prosthodontics is defined as the dental specialty pertaining to the diagnosis, treatment planning, rehabilitation, and maintenance of the oral function, comfort, appearance, and health of patients with clinical conditions associated with missing or deficient teeth and/or maxillofacial tissues using a biocompatible substitute [1], which is most commonly a prosthesis. In order for prosthesis to fulfill its function, it should be durable, aesthetic, accurate, and comfortable. These requirements should be accomplished by any prosthesis fabrication method.

Conventional fabrication methods involve recording an impression of the treatment site, pouring a stone model and constructing a wax pattern. The wax pattern is invested and replaced with a permanent material such as metal, ceramic, acrylic, or silicone. Such steps require considerable human intervention and manipulation of materials that may also exhibit inherent processing shrinkage and/or expansion [2, 3]. This can translate to increased processing errors and inaccuracies, as well as increased time and cost. Further, considerable skill is required to produce a prosthesis of good quality. The problems of the conventional protocol are however offset by the familiarity of the processes by the operators.

The limitations of the conventional fabrication method can be more obvious with prostheses that are fabricated less than commonly, such as facial prostheses that require a shade and features that match the remaining tissues [4–6]. Silicone facial prostheses fabricated using the conventional protocol also exhibit limited longevity from an aesthetic perspective because they are likely to tear at the thin margins and degrade on the external surface [7]. In general, the longevity of a facial prosthesis has been reported to be in the range of 6–12 months [8, 9]. To overcome this limitation, fabrication of multiple prostheses in one treatment episode has been advocated [4, 5]. Furthermore, due to the complexity of prosthesis construction and the limited expertise in specialist centers, patients tend to be deprived of adequate prosthetic treatment [10]. Reducing the reliance on the human variable and implementation automated design and fabrication techniques would therefore facilitate the production of more reliable prostheses.

Computer-aided design and computer-aided manufacturing (CAD/CAM) have revolutionized dentistry. With the continuous development of computerized engineering technology, digitized medical treatment modalities are becoming an integral approach for prosthodontics, orthodontics, and oral and maxillofacial surgery. This paper will review the existing computer-aided manufacturing streams for oral and maxillofacial prosthodontic treatment.

## 2. Digital Prosthodontic Treatments

As with any new technology, the utilization of computer-aided manufacturing in prosthodontics was not without impediments. Historically, the early application was crude and associated with compromised quality and precision of the prosthesis [11, 12]. Positively, the more recent literature reflects a tendency for continuous improvements of computer-aided manufacturing streams and a gradual shift towards wider acceptance of the new technology as a mainstream for prosthesis fabrication. From the practical perspective, the minimal requirement of computer-aided manufacturing is provision of a prosthesis that is, at least, equivalent to the one produced by conventional fabrication methods.

To date, the exponential increase in the application of computer-aided manufacturing in prosthodontics is attributed to continuous systems development and refinement, greater ability for quality control, parallel material development, and the possibility of virtual evaluation. Other areas of refinement include scanning technology, modelling software, and production systems, and the systems are becoming more user friendly [11, 12]. The development of quality control, production precision, and a simpler fabrication protocol by industry has tempted the medical profession to adopt and modify these technologies [11, 13–16]. In comparison to the conventional fabrication methods, computer-aided manufacturing has the advantage of omitting multiple error-introducing steps such as impression, waxing, and casting [11, 12, 17]. This is assumed to reduce the error sources and increase the precision of the prosthesis. Furthermore, since modelling and production are automated procedures, there is an overall reduction of fabrication time and cost.

Alongside computer-aided systems development, new materials have emerged for prostheses fabrication. Modern machines can utilize a broad array of metals, ceramics, and resins. Of most interest in prosthodontics are the high-strength ceramics (alumina and zirconia) that constitute a durable metal-free restoration material and can only be produced by computer-aided manufacturing [3, 18]. Prior to computer-aided manufacturing, metal-free restorations were prone to fracture and primarily reliable for single anterior tooth restorations. High-strength ceramics expand the indications for ceramic restorations to include multiunit prostheses and posterior teeth restorations.

A modified application of digital dentistry is the quantification of the effect of the proposed treatment prior to the active treatment phase. This takes advantage of the software precision in measurements and quantification. On the 3D models, volumes and distances can be precisely measured [19], and in the dental practice, analysis of tooth preparation can occur prior to prosthesis fabrication. In some instances, where the tooth preparation is not ideal or restoration thickness is minimal, modifications of the tooth preparation can be recommended to reduce the risk of mechanical failure of the prosthesis. Such feature can be coupled with a digital wax-up to ensure that any tooth preparation will facilitate the planned restoration [20]. Several authors have discussed models analysis and surveying for removable partial denture framework fabrication [21, 22]. This feature will locate the ideal path of insertion and abutment tooth undercuts and, subsequently, the ideal location of components and prosthesis design will be selected.

In prosthodontics, the early application of computer-aided manufacturing was to produce fixed prosthesis luted to teeth [13, 15, 23–25]. Systems have subsequently been developed to fabricate implant components and prostheses [17, 26]. In prosthodontics today, utilizing computerized technologies to fabricate prostheses is an acceptable treatment modality. Materials such as ceramics, metals (base metal alloys and titanium), resins, and waxes can be processed by the existing systems [12, 23, 26]. In addition, computerized technologies are used to plan and idealize surgical implant treatment [27–29]. Due to the precision that can be achieved with the aid of information from 3D digital radiographs, implant dimensions and placement locations can be determined using planning software without violation of critical anatomical features. Further, the need for bone and/or soft tissue grafting can be established.

A recent application of computer-aided manufacturing is the fabrication of removable prostheses. Removable partial denture metal frameworks can be produced directly from metal [21, 22, 30] or, alternatively, a resin pattern framework can be formed and then cast using conventional fabrication methods [31, 32]. Different computerized protocols have been proposed for the fabrication of complete denture bases [33–37] and are very useful for fabricating facial prosthesis [10], as the morphologies can be easily obtained by mirror image or average face form [38, 39] so that a more realistic and natural prosthesis can be manufactured [40]. A map of the surface morphology and colour can be saved virtually which facilitates future prosthesis fabrication. As extraoral scanning is a possibility, the whole experience will also be much more comfortable to the patient [40, 41]. The automated process will significantly reduce the reliance on technical skill and human variation. With the available systems, facial prosthesis can be produced from resin or wax [40–42]. Subsequently, it is invested and transformed to surgical grade silicone. In addition to prosthesis design, computerized planning also allows surgical idealizing of the defect site prior to prosthetic rehabilitation [10].

## 3. Computer-Aided Production Streams

Once the design of the prosthesis is completed by a computer-aided design (CAD) software, the data is transferred to a computer-aided manufacturing (CAM) software that controls the production unit. The aim of computer aided-manufacturing is to produce an accurate restoration with an accurate fit and correct morphology as designed by the CAD software. The following is a detailed discussion about subtractive and additive manufacturing. Special emphasis is placed on the materials effect and accuracy.

### 3.1. Subtractive Manufacturing

#### 3.1.1. Description

Subtractive manufacturing is based on milling the workpiece from a larger blank by a computer numeric controlled (CNC) machine. The CAM software automatically translates the CAD model into tool path for the CNC machine. This involves computation of the commands series that dictate the CNC milling, including sequencing, milling tools, and tool motion direction and magnitude [24]. Due to the unevenness of the features of dental restoration, the milling machines combine burs with different sizes. The accuracy of tool positioning has been reported to be within 10 *μ*m [24]. The CAM software also incorporates compensation steps for the cutter tool diameter which ensures that the milling bur reaches the desired surface without sacrificing necessary segment of the workpiece [24, 43].

**Figure 1:**
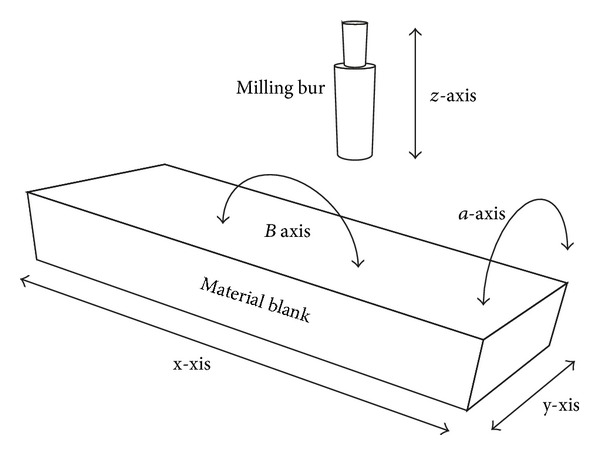
Schematic diagram illustrating the different milling axes. *x*-, *y*-, and *z*-axes are associated with 3-axis milling system. In addition to *x*-, *y*-, and *z*-axis, 4-axis milling system has an additional axis (*a*- axis). 5-axis milling system involves five axes (*x*-, *y*-, *z*-, *a*- and *b*-axes).

The dental CNC machines are composed of multiaxis milling devices to facilitate the 3D milling of dental work pieces (Figure 1). The 3-axis milling systems are the most commonly used in dental milling systems. In such systems, the milling burs move in three axes (*x*-, *y*- and *z*- axes) according to calculated path values. Therefore, 3-axis milling has the advantage of having minimal calculation and cumulative milling time [12]. In industry, 3-axis machines cannot produce convergence, divergence, and highly defined features or mill all the surfaces, unless the specimen is manually relocated. For the dental application, 180° rotation of the blank is incorporated within the machine, allowing 3D milling of the internal and external surfaces, producing divergence and convergence of the milled surfaces, and establishing greater definition of the surface features [12, 24]. Further, the speed of milling can be enhanced by incorporating two milling burs simultaneously. As the movement is restricted to the milling tool, large prosthesis cannot be produced by 3-axis machines.

In addition to the movements of 3-axis milling machines, the 4-axis machines allow for blank movements in an additional axis. This is a useful feature for milling a large blank and producing long span frameworks. The 5th axis of 5-axis machines is a rotating path of the milling tool or the blank. This facilitates production of very complex geometries and smooth external surfaces. The smooth surface is produced by the tangential movement of the milling bur. In industry, such machines are useful to manufacture very complex parts (e.g., curved holes). In dentistry, 5-axis machines are suitable for producing complex shapes such as acrylic denture bases [34]. For dental applications, the quality of the restoration is independent of the number of axes; instead, it reflects the method of processing the workpieces and CAD path of milling [12].

#### 3.1.2. Material

The materials processed by subtractive milling are metals, ceramics, resins, and waxes. A key advantage of milling is ensuring the durability of the workpiece since it is milled from an industrial grade blank. Milling can reduce fabrication flaws in dental prostheses, by relying more on the tighter quality control processing of the material manufacturer rather than commercial laboratory [44] so that manufacturing deficiencies, such as porosities and inhomogeneous consistency, are reduced [3, 17, 18].

Before computer-aided manufacturing, the clinical studies reported a reduced longevity of ceramic restorations compared to metal restorations. Initially, only glass ceramics were fabricated using conventional methods. Several studies showed that such materials are acceptable for inlay, onlay, or anterior crowns so long as they were cemented with resin [45, 46]. However, they exhibit a high failure rate in regions of high load such as posterior teeth [47]. Recently, highly dense ceramics with high flexural strength and fracture toughness were introduced [18]. In comparison to glass ceramics, zirconia can be used to fabricate dental prostheses such as fixed dental prostheses and implant abutments and frameworks where a high occlusal load is expected [3, 26, 48]. To produce the zirconia workpiece, the material should be heat treated to its melting temperature followed by strictly controlled cooling to ensure the zirconia is composed predominantly from the tetragonal phase [3, 49]. Such a set-up is not available in commercial dental laboratories. Instead, a large blank is industrially produced in a controlled environment and the dental workpieces are subsequently milled to the correct dimensions [18].

Two milling forms are available: hard machining and soft machining. The hard machining is used for metal, densely sintered zirconia, and composite resin while the soft machining is specifically used for presintered zirconia. Hard machining is based on milling the workpiece to its exact dimensions by a robust CNC system. Since it requires milling of blanks with high hardness, the machine has to be very strong to allow for application of heavy cutting forces and cutting power for efficient material removal. Therefore, the majority of the cutting power will turn into thermal energy and raise the temperature of the milling tool, which can reduce its life. Further, the surface temperature rise will be accentuated if the milled material is of low thermal conductivity (e.g., titanium and zirconia) [50, 51]. Thus, constant cooling is required to prevent overheating of the milled material [50]. Due to the brittle nature of ceramics, it is very likely that it will be more affected than metal [44]. Subcritical ceramic surface damage could develop in the form of surface microfractures, chipping defects, and altered surface quality [51, 52] and could constitute a point for crack propagation within the restoration under occlusal forces [52]. The extent of the damage is dependent on the material of the workpiece [53] and ranges from 15 to 60 *μ*m [54–56]. To reduce such complications, milling is better accomplished in two steps: a first rough milling is done at a low feed rate and high cutting force while the final fine milling is performed at a higher feed rate and reduced cutting forces [18, 44]. The fine milling will reduce the chip thickness and minimise surface roughness [57–59]. In relation to zirconia milling, common disadvantages of hard machining are that it is time consuming and that the zirconia phase transformation to monoclinic phase occurs [18, 44, 60]. Such a phase transformation will exacerbate surface microcracking and low thermal degradation.

Consequently, soft machining or dry processing has been adopted by several manufacturers for the purpose of simplifying the milling and to improve time efficiency. Soft machining is based on the milling of an oversized piece of a presintered zirconia workpiece followed by sintering. The milled zirconia is at the presintered state and the composition differs from the hard machined zirconia to reduce its hardness which enhances the machinability. The sintering procedure will cause about 25–30% shrinkage of the workpiece [18]. This approach has the advantages of quicker milling, reduce cutting forces, increase the tool life, potentially better surface quality, and no moisture absorption of the zirconia blanks which omits the need for drying the milled zirconia prior to sintering [3, 12, 18]. Further, as the milled presintered zirconia will be sintered, it will have a more consistent tetragonal phase on the surface than hard machined zirconia. The risk with this procedure is the increased dimensional discrepancy in comparison to hard machined zirconia which has been reported in laboratory and clinical studies [61, 62]. Nevertheless, the continuous improvements in soft machining of zirconia reflect a now reliable shrinkage compensation mechanism [63].

Clinically, although there are several advantages of zirconia, it exhibits inherent limitations such as a high incidence of ceramic veneer fracture and material instability [18, 61]. In order to reduce such complications, different strategies have been proposed. The zirconia should be produced with accurate dimensions so the need for manual adjustment is eliminated. Postsintering adjustment can lead to phase transformation defect introduction to the zirconia and subsequent weakening of the zirconia framework [3, 18, 44, 64]. Several authors have reported increasing the zirconia thickness to enhance its clinical durability [65–68]. This could be easily incorporated with digital production systems. Full customization of the zirconia framework will ensure maximal bulkiness of the material that has been shown to enhance the durability of a zirconia prosthesis [69, 70]. In addition, it will provide more support to the veneering ceramic or reduce the thickness of the veneering ceramic that reduces the possibility of ceramic chipping [65, 66]. More recently, monolithic zirconia prostheses have been proposed where the prosthesis is entirely fabricated from zirconia. Such treatment modality appears to be promising for tooth-supported prostheses [71] and implant-supported prostheses [72]. This appears to be coming from a more popular option especially with the increased application of stained or translucent zirconia that overcomes the problem of high value of zirconia [73, 74].

In vitro studies have confirmed that zirconia prostheses are not as durable as metal prostheses. This is true for tooth-supported prostheses and implant-supported prostheses [75, 76]. However, it has also been confirmed that the durability of zirconia prostheses is within a clinically acceptable range to withstand physiological occlusal forces. This is supported by clinical studies that show that substructures made from zirconia are suitable for copings, fixed dental prostheses, implant abutments, and implant frameworks [77–81].

#### 3.1.3. Accuracy

As stated earlier, milling is anticipated to eliminate waxing, investing, and casting of prostheses which is assumed to improve the overall precision. However, there is a lack of compelling evidence supporting this assumption for tooth-supported restorations [82, 83], because, in terms of fit, there is an overall tendency for the restorations produced by conventional methods to exhibit better fit than milled restorations. This applies to milled metal [83, 84] and ceramic restorations [85]. In relation to milled titanium, Tan et al. found that milled titanium crowns exhibited a vertical gap of 79.4 *μ*m, while cast noble metal crowns had vertical opening of 23.9 *μ*m [83]. In a similar study, Han et al. compared the marginal accuracy of milled titanium and cast titanium crowns [84]. There was a tendency for a smaller marginal opening for cast titanium (52–76 *μ*m) than CAD/CAM titanium (60–80 *μ*m). In relation to lithium disilicate, milled ceramic veneers tended to exhibit horizontal (231.0 *μ*m) and vertical (545.8 *μ*m) gaps twice those of pressed veneers (horizontal gap = 105.6 *μ*m and vertical gap = 242.4 *μ*m) [85]. This difference was significant enough to be associated with more microleakage. A systematic review revealed that more fit discrepancies of zirconia frameworks were associated with soft machining, prosthesis curvature, and span length [82].

However, fit problems might not be purely from milling. Two factors could explain the limited accuracy of the CAD/CAM protocols. The first factor is related to the familiarity and the associated learning curve in relation to restoration fabrication following the CAD/CAM protocols compared with the well-established conventional protocol. The second factor is although multiple steps are omitted, the CAD/CAM protocol introduces additional steps in the fabrication process which may result in the introduction of inaccuracies. Steps such as scanning, locating the margin digitally, software modelling, and milling will inevitably introduce source of inaccuracy [51, 86].

**Figure 2:**
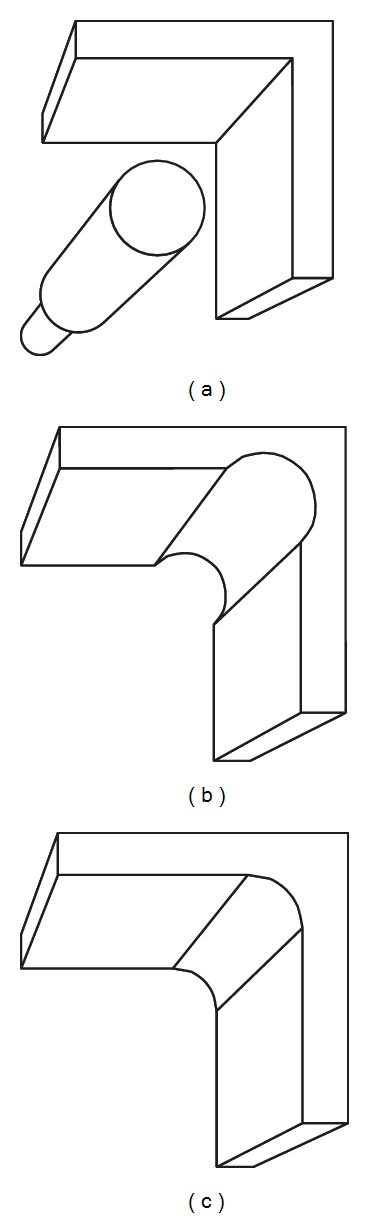
The effect of bur diameter in internal line angle production. (a) Sharp virtual line angle cannot be produced by rounded bur. Therefore, internal surface inaccuracy will occur on the milled restoration in the form of (b) negative error after overmilling of the sharp corner or (c) positive error after undermilling of the sharp corner.

**Figure 3:**
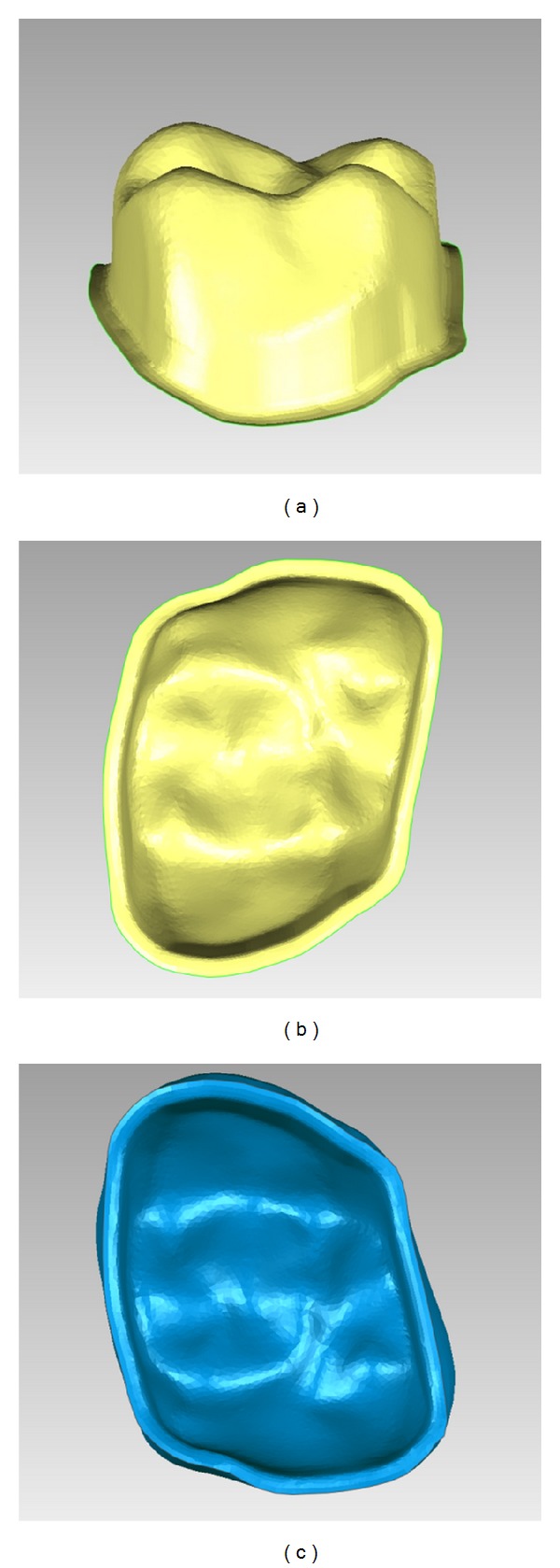
(a) Side and (b) occlusal image of prepared tooth illustrating smooth and continuous surfaces and well-defined preparation margin. Such preparation features will enhance the accurate milling of the final restoration. (c) The rounded features of the virtual restoration fitting surface can easily accommodate the milling burs.

The production of the fine details by milling is largely dependent on the diameter of the smallest milling bur which is normally about 1 mm [12, 24, 43]; however, smaller diameter milling burs do not appear to produce fine detail for accuracy [86, 87]. Örtorp et al. reported that, in order to mill the internal angle with a diameter less than the diameter of the smallest fitting bur, a drill compensation feature has to be incorporated within the software to provide room for the bur movement (Figure 2) [43]. When used, however, this feature was found to produce negative fit errors and dramatically increase the internal space between the restoration and the prepared tooth surface. Excessive cement space has been attributed to a compromise in the mechanical durability of a restoration [88]. Excessive cement space also results in a loose fitting restoration that may affect the accuracy of seating the restoration, resulting in occlusal interferences, horizontal marginal discrepancies, and a loss in retention of the restoration [89]. Further, in cases with limited occlusal space, the final restoration thickness will be reduced, which increases the susceptibility of a mechanical failure. If cement space is not incorporated, however, positive errors will be produced and the restoration will not fit unless manually modified. Problems created by incorrect cement space can be minimised by incorporating rounded line angles on the tooth preparation. Doing so will minimise the impact of the drill compensation as the edge diameter can accommodate the bur diameter [3, 12]. In addition, a smooth and continuous preparation surface with minimal irregularities and well-defined preparation margins will enhance the quality of the milled restoration (Figure 3).

Milling accuracy is dictated by materials properties. High material hardness means low machinability and more involved forces [90]. Titanium and densely sintered zirconia are difficult to machine, rendering the bur more susceptible to tool failure and wear [50, 90–92]. Consequently, the internal surface might be under-milled, hindering the fit of the restoration [43]. In addition, the hardness of these materials means that they are more prone to surface chipping and chattering especially under high feed rates, high cutting speed, and deficient cooling [91, 93]. These cutting conditions also cause excessive vibrations and exert thermal and mechanical stresses on the workpiece, which contributes to dimensional distortions, especially in thin edges [94]. To overcome these limitations, regular maintenance and bur replacement have been advised [43].

A clinically acceptable margin is within 100 *μ*m [95, 96], and while there is a significant difference reported in the laboratory studies, these may not be of clinical significance. It is worth noting however that an “adaptation step” was included in many of the studies where the investigators manually modified the internal surface of the CAD/CAM framework with a fine grit size diamond bur to eliminate internal premature contacts [62, 97–99]. The rationale behind this manual refinement was to eliminate internal binding surfaces which prevented adequate seating at the crown margin allowing a better marginal fit to be obtained [100]. Although this step clearly enhances the fit of the final restoration, it also indicates a deficiency of the CAD/CAM systems in producing accurately fitting restorations.

On the contrary, implant milled prostheses were consistently better fitting than conventionally fabricated implant prostheses [101, 102]. This observation was correct for components fabricated from metal and ceramic. The vertical gap for milled titanium and zirconia abutments was reported to be in the range of 2.5–3.2 *μ*m [103]. Further, the rotational movement was reported to be minimal (less than 3°) [104]. In relation to implant frameworks, milling consistently provides very accurately fitting frameworks with a vertical fit in the range of 1 to 27 *μ*m, and the influence of prosthesis span appears to be minimal [105, 106], which is opposite to the observation for cast metal frameworks [107]. Milled abutments have also been shown to have minimal roughness with a well-defined geometry edge and a vertical gap of 0.7 *μ*m. This is superior to abutments produced by selective laser melting (vertical gap = 11.3 *μ*m) or casting (vertical gap = 9.1 *μ*m) [108].

The superior accuracy of milled implant components compared to tooth-supported restorations could be due to the simple and accurate numerical representation of the geometry of the implant components. This reduces the reliance on the scanning function to register the location and orientation of the implants, and no milling compensation feature is required for the implant framework or abutment [17].

### 3.2. Additive Manufacturing

#### 3.2.1. Description

Additive manufacturing systems have recently been introduced as a method to construct dental restorations and medical devices. Additive manufacturing is defined as the process of joining materials to make objects from 3D model data, usually layer upon layer [10, 16, 109]. Once the CAD design is finalized, it is segmented into multislice images. For each millimetre of material, there are 5–20 layers, which the machine lays down as successive layers of liquid or powder material that are fused to create the final shape. This is followed by workpiece refinement to remove the excess materials and supporting arms. Similar to the subtractive systems, a form of CNC machine is used with a processing head that moves in two axes (*x*- and *z*-axes) and the specimen platform or the processing head moves in the vertical axis (*y*-axis) [110, 111].

Originally, the additive manufacturing methods were implemented to fabricate prototype models and patterns with reliable accuracy and repeatability that could be produced in a short time. In prosthodontics, additive manufacturing can fabricate a preproduction pattern (wax or plastic) that can be transformed to a definitive prosthesis, and it can directly produce definitive workpieces in metals, resins, or ceramics [10, 16, 109]. The application of additive manufacturing in dentistry is due to its ability to produce a variety of shapes that conform to any biological site. The additive systems used in dentistry are stereolithography, selective laser sintering or melting, and 3D printing. Regardless of the method, all share the following features that distinguish them from subtractive manufacturing:

- incremental vertical object build-up
- no material wastage
- large objects produced
- passive production (i.e., no force application)
- fine details production.

**Figure 4:**
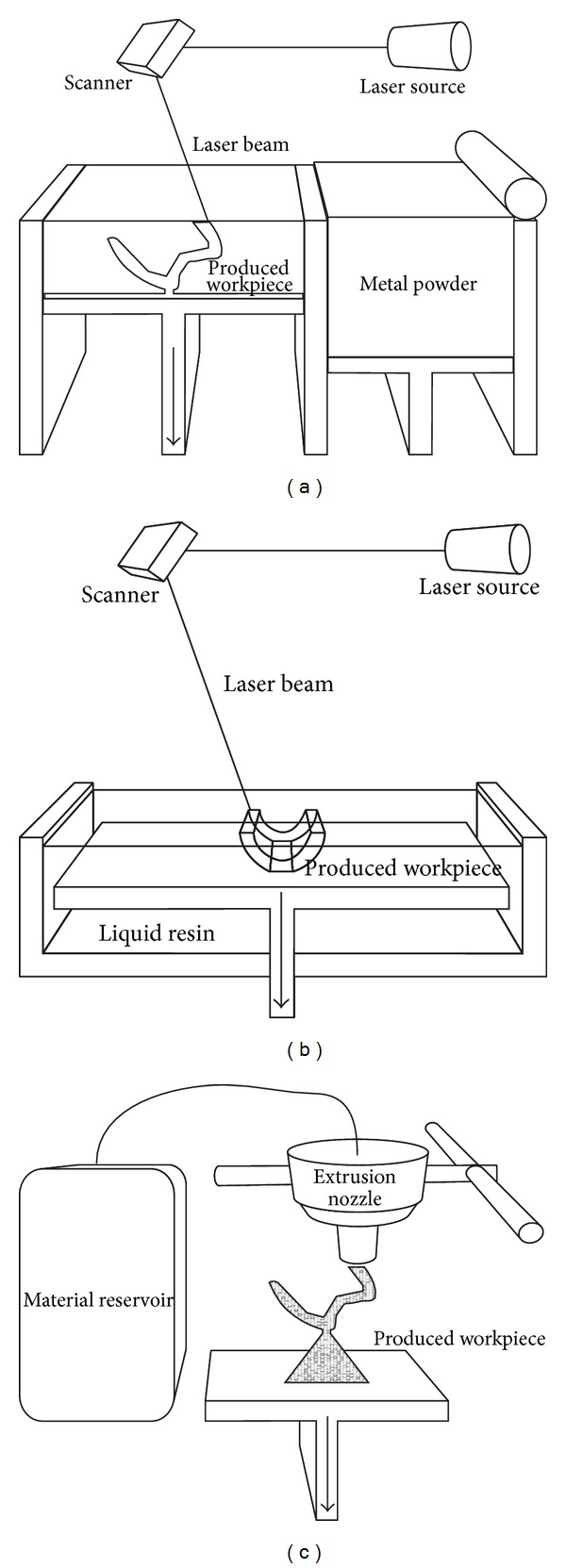
Schematic diagrams of the different additive manufacturing apparatus. (a) Selective laser melting. (b) Stereolithography. (c) 3D printing. The arrows indicate the direction of platform movement.

Selective laser sintering or selective laser melting produces a 3D model by laser sintering or melting a powder, layer by layer using a laser beam (Figure 4(a)). The laser beam locally raises the temperature close to the melting point of the metal particle, to avoid complete melting [112, 113]. The platform is slightly immersed in the powder, and powder thickness is controlled by a cylinder rolling on the powder pool. After each new powder layer application, the laser melting process is repeated until the 3D object is completed. Oxidation of the metal can be controlled by confining the melting to a sealed gas chamber. The term selective laser sintering is used to describe the fabrication of a pattern from ceramics or polymers while selective laser melting describes pattern fabrication from metal [16]. Selective laser melting is the only additive method that is available to produce metal workpieces such as crowns, fixed dental prostheses, or removable partial denture frameworks. Further, this technique can produce customized implants for maxillofacial applications or joint replacement.

Stereolithography produces the solid layers using a concentrated ultraviolet light beam that moves on a curable liquid polymer pool (Figure 4(b)). As the first layer is polymerized, a platform is lowered a few microns and the next layer is cured. This process is repeated until the whole solid object is completed. The object is then rinsed with a solvent and placed in an ultraviolet oven to thoroughly cure the resin. In dentistry, stereolithography is routinely used to produce resin objects such as surgical templates for oral and extraoral implant placement and preprosthetic surgery. Additional applications of stereolithography are the fabrication of facial prosthesis patterns, occlusal splints [114], burnout resin patterns [115], and investing flasks [116]. With the aid of multislice CT data, real size anatomical models of a patient can also be replicated to facilitate visualization of bone anatomy [117]. Further, these anatomical models can be used to assist with the fabrication of customized implants for hard tissue reconstruction.

3D printing extrudes material from a nozzle that solidifies as soon as it is deposited on the manufacturing platform (Figure 4(c)). The layer pattern is achieved through horizontal nozzle movement and interrupted material flow. This is followed by vertical movement for the sequential layer deposition. There are a range of materials that can be used for 3D printing. This includes thermoplastic materials, such as waxes, resins, or fused filament, which pass through a heated nozzle and solidifies immediately after extrusion. Alternatively, liquid ceramic or resin materials with a binder can be printed [35, 118], which, following deposition, solidifies immediately [119, 120]. Some systems also allow for multicolour production [118]. This approach is used in dentistry to fabricate dental models, facial prosthesis patterns, acrylic prostheses, investing flasks, and castable or ceramic frameworks [35, 118]. 3D printing is distinguished from other fabrication methods in the ability to print multiple materials at one time [119].

#### 3.2.2. Material

Material selection depends on the purpose of the prosthesis and the manufacturing procedure. In comparison to subtractive processing, this method is more economical since it does not result in any material wastage, and any unused material is completely reusable for future processing [10, 109]. In addition, there is minimal restriction on the ability to fabricate large workpieces (e.g., facial prosthesis and skeleton models), which is not the case with subtractive methods that are more suitable for smaller workpieces [40–42]. Additive manufacturing also allows the fabrication of workpieces with different consistencies and material properties [121].

**Figure 5:**
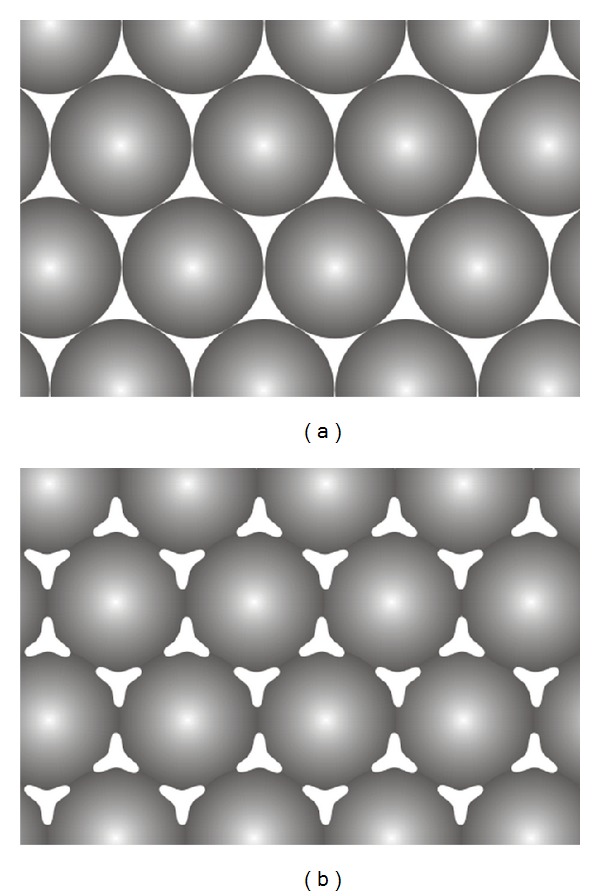
The effect of selective laser melting of metal powder. (a) Premelted metal particles. (b) Postmelting figure illustrates the necks formation between the particles.

Some prostheses serve for aesthetic purposes, such as facial prostheses, and significant durability is not a requirement. Instead, rapid manufacturing is beneficial as it allows the production of several prosthesis duplicates. In comparison, for an intraoral prosthesis to be functional, it should be durable enough to withstand occlusal loads. It is well reported that the metal pattern produced by selective laser melting exhibits microporosities, in the range of 30–45% [113, 121]. Porosity was also observed in the dental frameworks produced by selective laser melting [22] and has been attributed to the selective melting process [113, 122]. As laser melting takes place, the external surface of the metal transforms to a liquid phase. The liquidised surface flows and fills the pores between the metal particles and solidifies to form a continuous solid phase. Greater liquid flow between the metal particles and lower initial porosity results in the production of a low porosity microstructure. The selective melting process should not, however, completely melt the metal particles; otherwise, the melted particles will aggregate and form larger spheres [112, 113, 122], resulting in major dimensional discrepancies in the final workpiece [112, 113]. To avoid this, the metal particles should be heated to just below the melting temperature to ensure melting is confined to the external surface of the particles and fusion contact forms necks between the adjacent powder particles (Figure 5).

Reducing the porosity is desirable as it will increase the tensile strength of the framework [112, 113]. Certain parameters can be modified by the manufacturer. For example, smaller particle sizes, greater loose powder density, higher laser intensity, reduced scan speed, and smaller layer thicknesses will contribute to increased product density. However, this should be weighed against the potential risk for increased dimensional error, as greater laser power and a lower scan speed can result in greater distortion [112]. Continuous improvements appear to be promising and high metal density is achievable with ideal manufacturing parameters [123].

Porosity was reported to be advantageous for implant fabrication as it produces implants with similar elasticity to bone [124]. Further, the porous structure facilitates ingrowth of bone that can promote osseointegration [125, 126]. However, such advantages should be accepted with caution as the mechanical durability could be reduced considerably [121]. This is especially critical for intraoral prostheses where mechanical durability is necessary for the prosthesis to withstand occlusal loads. Nevertheless, a recent short-term clinical study reported promising outcomes for posterior crowns [127]. Multiunit prostheses are subjected to considerably greater tensile forces and clinical studies are required to support the clinical validity of this manufacturing technique. To overcome problems of the porosity, Wu et al. utilized additive manufacturing to produce a wax pattern that was eventually cast using the conventional technique [128].

Recently, high-strength zirconia frameworks have been produced by 3D printing [119, 120, 129, 130]. This novel fabrication method is thought to overcome the problems created by milling, such as surface cracking, shrinkage, and material wastage. The zirconia framework is printed from a suspension of nanoscale zirconia particles with an inkjet printer [119, 120]. Initially, the printed shape is maintained by drying, but the final strength is reached by sintering [119, 120].

The reported strength was of the zirconia prosthesis was 764 MPa and the fracture toughness was 6.7 MPam^0.5^ [120]. SEM imaging of the printed and sintered zirconia revealed a homogeneous microstructure; however, submicron-sized pores were also detected and attributed to the clogging of nozzles during the injection of zirconia paste. These defects were found to reduce the strength of zirconia frameworks. It is thought that this problem could be overcome with advances in printing hardware [120].

#### 3.2.3. Accuracy

**Figure 6:**
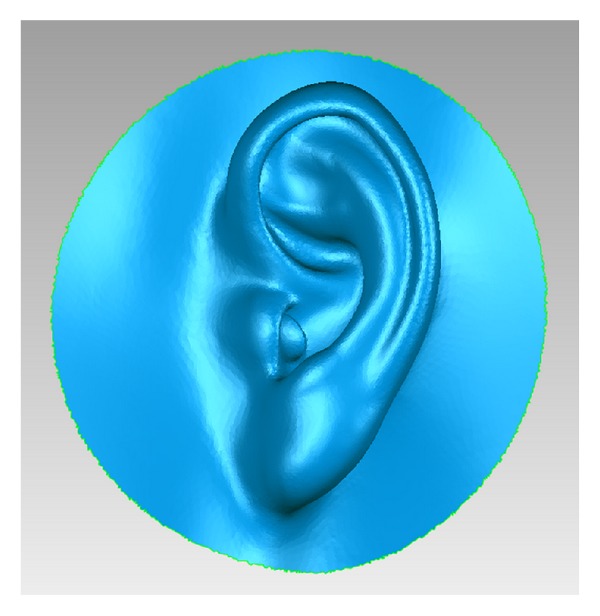
Complex facial features such as corrugation, corners, or tubes can be easily produced by additive manufacturing.

**Figure 7:**
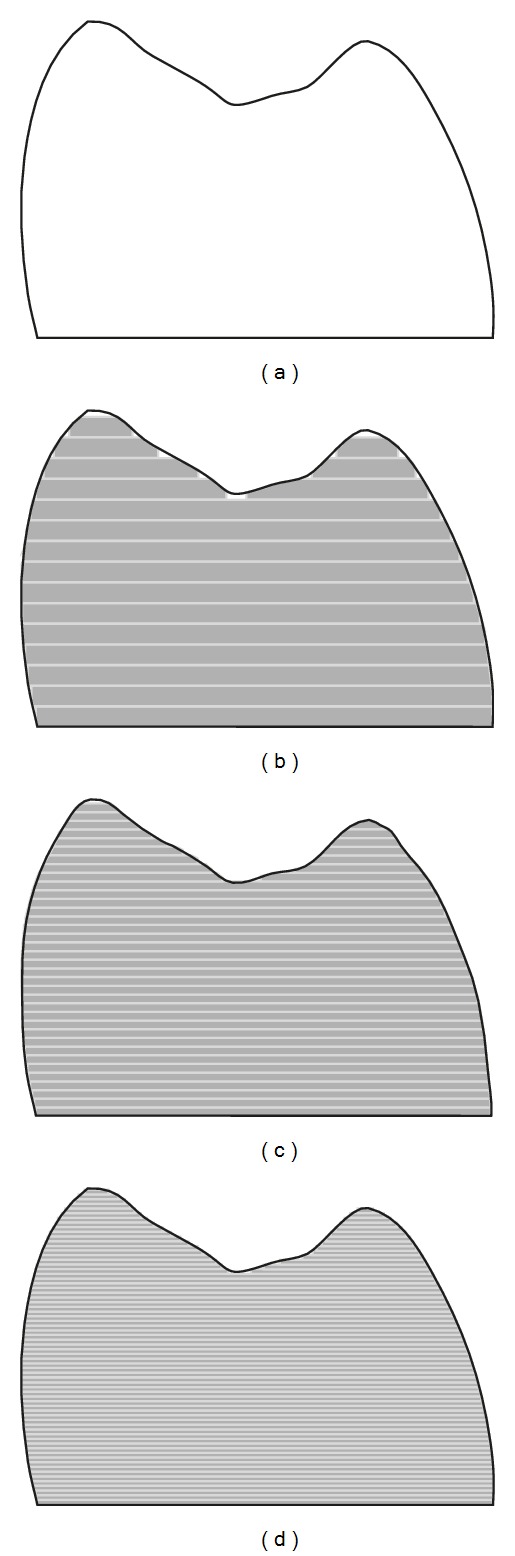
The effect of layered production on the surface accuracy. (a) Smooth surface is ideal for dental restorations. (b) Thick layers will increase the prominence of surface stepping. (c) and (d) As the layers thickness is reduced, the surface accuracy will increase. The corrugated surface (occlusal surface) is more affected by the steps than the vertical surfaces.

Among the advantages of additive manufacturing is the ability to produce customized workpieces that fit patient hard and/or soft tissues [10, 109]. The workpieces can include detailed morphology, sharp corners, undercuts, or voids. Such features may be desirable for facial prostheses (Figure 6). Because no drilling tool is involved, no compensation feature is required as is necessary for the subtractive manufacturing. Further, the whole production process is passive and involves no force application. However, due to the production procedure, which involves sequential layering, the external surface tends to have stepped and coarse morphology representing each fabrication layer along the construction direction [110]. Such stepping adversely affects the surface texture and the overall dimensional accuracy of the workpiece [110], which could be a problem clinically if the prosthesis is not polished or veneered [22, 31] (Figure 7). The vertical walls were minimally affected by stepping while the corrugated or sloping surfaces are more prominently influenced [123]. Therefore, concerns have also been raised regarding the accuracy of the occlusal surface of prostheses produced using this technique [119]. The accuracy of additive technique is dependent on layer thickness and the width of curing beam. The thinner the layers and the narrower the curing beam, the more accurate the final product; however, increasing the number of layers and reducing the diameter of the beam will exponentially increase the fabrication time [110, 112, 113].

In the dental literature, there are a limited number of studies that have evaluated the accuracy of prostheses fabricated by additive manufacturing. In relation to selective laser melting, the dimensional accuracy of metal workpieces has been reported to be in the range of 3–82 *μ*m, which is clinically suitable for intraoral prosthesis [113, 123, 131]. Accuracy can be adjusted by controlling particle diameter (30 µm) and layer thickness (50–200 *μ*m each) [113, 122]. The smaller the dimensions, the greater the accuracy and the density of the final product. Increasing laser intensity and melting time is desirable to increase the density of the workpiece, but this should be weighed against the increase in dimension error that can occur as a result [112]. Although the distortion of each layer is minimal, the accumulated error for all the layers can cause a measurable error [132]. The manufacturer should therefore control the processing parameters, to ensure ideal parameters for a given application [113].

In dentistry, the accuracy of selective laser melting production has been evaluated primarily by assessing the fit of the dental prosthesis. Quante et al. found that the marginal fit of crown copings produced by selective laser melting of noble metal alloy and base metal was in the range of 67 to 99 µm which is within the acceptable clinical range [133]. In the same study, the copings of the two alloys were minimally affected by ceramic veneer application. This suggests that alloy selection has minimal influence on the accuracy of selective laser melting. Ucar et al. found that the fit of laser-melted base metal alloy copings is comparable to the fit of cast base metal alloy copings [134], and Örtorp et al. showed that selective laser melting produced fixed dental prosthesis frameworks with almost half the fit discrepancies (84 *μ*m) of those produced by milling (166 *μ*m) [43]. The latter study also observed a uniform internal fitting surface when compared to milled frameworks that was attributed to the absence of a compensation mechanism in the production process. This outcome was confirmed by Castillo-Oyague et al. who found that copings produced by selective laser melting exhibited half the vertical gap (25 *μ*m) of cast copings [135]. The overall dimensional accuracy of selective laser melting has been attributed to the lack of force application and vibration of the machine during production of the workpiece. This feature is of significant importance as it allows the production of delicate and thin structures without causing deformation or recoil of the components. For example, removable partial denture framework components can only be produced by selective laser melting [22, 136]. Williams et al. also reported that the fit of removable partial denture frameworks produced using the additive manufacturing procedure is comparable to frameworks produced using conventional methods [22].

Although the fit of tooth-supported frameworks produced by selective laser melting may be better than those produced by milling [43], similar findings have not been observed for implant components. Implant abutments produced by selective laser melting have been shown to exhibit greater surface roughness and microgaps compared to machined or cast abutments [108]. Further, the geometry of abutments produced by selective laser melting was blurred in comparison with the sharply defined connection of the milled components. Although the inaccuracies generated by selective laser melting (11 *μ*m vertical gap) still reside within the clinical acceptability range, it reflects system limitations where future improvements are very desirable.

For stereolithography, the range of each layer thickness is 50–150 *μ*m [137]. In dentistry, this technique is primarily used to produce surgical implant guides. The accuracy of intraoral implant placement positions has been evaluated to provide an indication of the benefit of surgical implant guides produced by stereolithography and has been found to be in the range of 0.4–2.0 mm, and angulations are in the range of 2–5° [27, 29, 138]. Similarly, extraoral implant placement accuracy was 1.5 mm [139]. Farley reported that some stereolithography templates required intraoral relining to improve the fit on the adjacent teeth [28]; however, this discrepancy could not be related to manufacturing process. Instead, clinical variability such as soft tissue fit and compressibility and implant insertion in less dense bone may be the main source of the discrepancies [27, 139]. Distortions will also occur in the construction of 3D images from multislice radiographs. Despite this, the discrepancies observed endorse the clinical recommendation that these techniques do not eliminate the importance of surgeon experience, awareness of critical anatomical features, and the maintenance of a safe zone of 2 mm from critical features such as adjacent teeth when planning implant placement [29]. Overall, the accuracy of the surgical guides could be improved by fabricating them to fit the alveolar bone instead of soft tissue and by the use of fixation screws [27, 29]. Nevertheless, a split mouth clinical study revealed that the stereolithography guides allowed for implant placement closer to the planned position than conventionally fabricated guides [28].

In general, stereolithography provides good overall contour of facial prosthesis, with several authors reporting well-fitting facial prostheses fabricated by stereolithography [41]. The range of accuracy of the facial prosthesis pattern fabricated by this method resides in the range of 0.1–0.4 mm [7, 140].

Salmi et al. reported the dimensional accuracy of occlusal splits fabricated by stereolithography to be 0.3 mm [114]; however, true quantification of fit was not conducted. Overall, the margins were less than ideal which could be due to the stepping surface feature [40, 42]. Some authors have suggested wax relining prior to investing the pattern to compensate for these inaccuracies [40]; however. with regular advances in development of the software and hardware, it is very likely that this problem is of minimal impact.

There is very limited data on the accuracy of 3D printing for dental applications. Ebert et al. reported that this method allows the fabrication of very accurate ceramic workpieces [120], and the production of sections of 100 *μ*m is feasible. Silva et al. reported that the tolerance of the fabricated workpiece is less than 25 *μ*m, which is very acceptable for intraoral application [119]. In comparison, an evaluation of the dimensional errors of printed dentures found a mean deviation of 5 *μ*m, but dimensional distortions of up to several 100 *μ*m were detected [118]. As the 3D printing of dental prosthesis is still in its infancy, it is very likely that significant quality improvement will occur in the future making this technology very competitive with the existing fabrication methods [119].

## 4. Conclusions

Computer-aided manufacturing continues to undergo significant and regular improvements so that it is very likely that, in the near future, wide acceptance of its use in dentistry will occur. Currently, subtractive milling is the most widely implemented computer-aided manufacturing protocol in dentistry and it has been shown to be a suitable method for fabricating intraoral prostheses. Additive manufacturing is currently an exponentially growing fabrication method and will most likely be used more frequently in dentistry in the future as its accuracy and range of applications develop. In terms of material processing, both techniques introduce material defects. The subtractive methods, however, currently produce more homogenous objects making this method more suitable for the production of intraoral prosthesis that can withstand higher occlusal loads. Additive methods have the advantage of producing large objects, with surface irregularities, undercuts, voids, and hollow morphology that makes them suitable for manufacturing facial prostheses and metal removable partial denture frameworks. Computer-aided manufacturing procedures will indisputably change many aspects of dentistry in the future, particularly in relation to treatment simplicity and production time. It is therefore critical for clinicians and technicians to be familiar with the advantages and disadvantages of computer-aided manufacturing as these procedures continue to develop and become an integrated part of dentistry.
